# Targeting aldehyde dehydrogenase for prostate cancer therapies

**DOI:** 10.3389/fonc.2022.1006340

**Published:** 2022-10-10

**Authors:** Miao Ma, Wenyou He, Keyu Zhao, Linyuan Xue, Siyuan Xia, Baotong Zhang

**Affiliations:** Department of Human Cell Biology and Genetics, School of Medicine, Southern University of Science and Technology, Shenzhen, China

**Keywords:** prostate cancer, aldehyde dehydrogenase, targeted therapy, drug resistance, cancer stem-like cells (CSCs), signaling transduction

## Abstract

Prostate cancer (PCa) is the most common cancer in men in the United States. About 10 – 20% of PCa progress to castration-resistant PCa (CRPC), which is accompanied by metastasis and therapeutic resistance. Aldehyde dehydrogenase (ALDH) is famous as a marker of cancer stem-like cells in different cancer types, including PCa. Generally, ALDHs catalyze aldehyde oxidation into less toxic carboxylic acids and give cancers a survival advantage by reducing oxidative stress caused by aldehyde accumulation. In PCa, the expression of ALDHs is associated with a higher tumor stage and more lymph node metastasis. Functionally, increased ALDH activity makes PCa cells gain more capabilities in self-renewal and metastasis and reduces the sensitivity to castration and radiotherapy. Therefore, it is promising to target ALDH or ALDH^high^ cells to eradicate PCa. However, challenges remain in moving the ALDH inhibitors to PCa therapy, potentially due to the toxicity of pan-ALDH inhibitors, the redundancy of ALDH isoforms, and the lack of explicit understanding of the metabolic signaling transduction details. For targeting PCa stem-like cells (PCSCs), different regulators have been revealed in ALDH^high^ cells to control cell proliferation and tumorigenicity. ALDH rewires essential signaling transduction in PCa cells. It has been shown that ALDHs produce retinoic acid (RA), bind with androgen, and modulate diverse signaling. This review summarizes and discusses the pathways directly modulated by ALDHs, the crucial regulators that control the activities of ALDH^high^ PCSCs, and the recent progress of ALDH targeted therapies in PCa. These efforts will provide insight into improving ALDH-targeted treatment.

## Introduction

Human prostate cancer (PCa) is the most common cancer and the second leading cause of death in men in the United States. There will be 268,490 new cases and 34,500 deaths from PCa in the United States in 2022, with PCa alone accounting for 27% of all cancer diagnoses (1). Although many new advances in research on PCa, the underlying molecular mechanism is not fully understood. Generally, localized PCa can be removed by radiation therapy or radical prostatectomy. Androgen deprivation therapy is effective as androgen receptor signaling is a dominant pathway that fuels PCa progression (2–4). About 10 – 20% PCa progresses to its advanced stage, castration-resistant prostate cancer (CRPC) (5–7). Most CRPC typically gains capabilities in metastasis and resistance to systematic therapeutics and thus is lethal to the patients (8, 9). Currently, PARP inhibitors are the only FDA-approved targeted therapy for PCa, and other effective drug targets are under development.

Heterogeneity is crucial to the sensitivity of treatment for PCa, and emerging evidence has shown that there are subsets of PCa cells with stem cell properties in the tumor microenvironment (TME) (10). These prostate cancer stem cells (PCSCs) are responsible for PCa initiation, progression, therapy resistance, and metastasis (11–16). In addition, PCSCs have the characteristics of self-renewal and resistance to radiation or chemotherapy (13, 14, 16, 17), and thus are effective targets for PCa treatment potentially (11, 16, 18). For instance, retinoic acid was used in clinical trials to drive the differentiation of PCSCs ^80^.

Aldehyde dehydrogenase (ALDH) is famous as a marker of cancer stem cells in different cancer types, including PCa (19). Generally, the ALDHs are a superfamily with 19 different isoforms in humans and catalyze aldehyde oxidation into less toxic carboxylic acids (18). The expression of ALDH is elevated in PCSCs. It has been shown that ALDH^high^CD44^+^ PCa cells and the corresponding ALDH^low^CD44^−^PCa cells manifest as PCSCs and non-PCSCs (20). A recent review by Dr. Jakob Püschel et al. summarized the roles of different members in ALDH superfamily in PCSCs detailly (18). Among them, ALDH1A1 and ALDH3A1 are the most reported because they play a critical role in maintaining PCSC stemness (18, 21, 22). On the other hand, Dr. Saketh S. Dinavahi et al. give us a detailed review of the latest progress on ALDH inhibitors in cancer therapy (23). Several efforts have been made to understand particular signaling transduction and metabolic pathways in ALDH^high^ cells. Moreover, ALDH is more than a marker of cancer stem-like cells, it also directly regulates diverse signaling pathways. This review will focus on the ALDH directly involved signaling pathways, the regulators of ALDH^high^ PCSCs, and the potential strategies to overcome the limitations of ALDH-targeted therapy in PCa.

## ALDH-associated metabolism and convergence on signaling pathways

**Figure 1:**
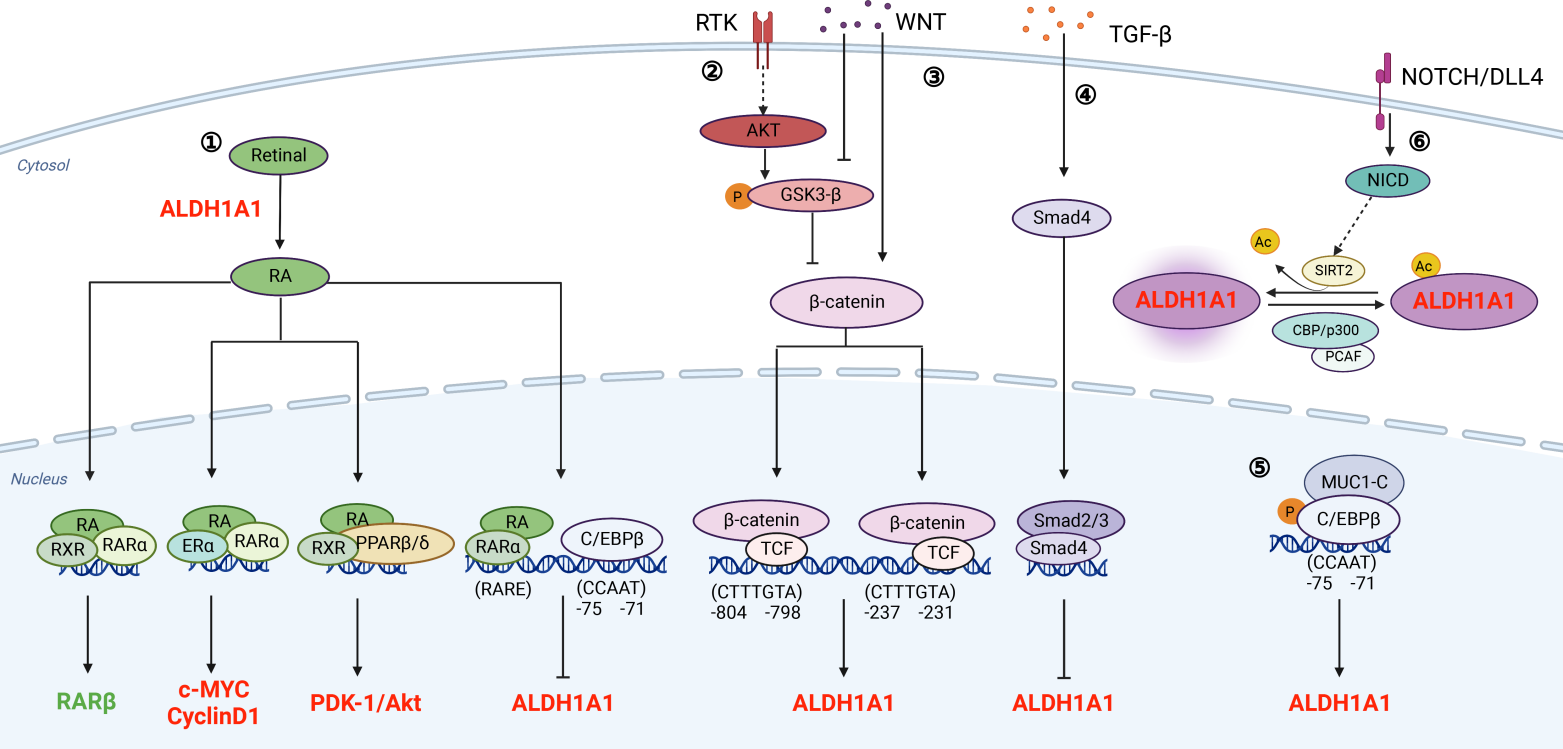
Signaling pathways that associate with ALDHs directly.① RA signaling pathway: ALDHs oxidize retinal to generate RA. Meanwhile, the transcription of ALDH is also regulated by RA. As a feedback loop, increased levels of ALDH promote the synthesis of RA; ② and ③ PI3K/AKT pathway and WNT signaling pathway regulate the transcription of ALDH through β-catenin; ④ TGF-β signaling pathway: TGF-β inhibits the transcription of ALDH through Smad4; ⑤ MUCI-C pathway: The protein complex of MUC1-C and C/EBPβ binds to the promoter of ALDH and promotes its transcription; ⑥ NOTCH/DLL4 pathway: Notch signaling promotes ALDH activity by inducing SIRT2 and triggering ALDH1A1 deacetylation. The regulators in red refer to those promoting PCSCs, and the regulators in green refer to those inhibiting PCSCs. This figure is generated by BioRender with an agreement number of QR24HA6NER.

ALDHs typically catalyze aldehyde oxidation into less toxic carboxylic acids (18). Nevertheless, ALDHs are not only biomarkers of cancer stem-like cells but also directly participate into signaling transduction which regulates biological activities of cancer cells. Herein, we summarize the well-recognized pathways involving ALDHs directly (**Figure 1**).

### Retinoic acid (RA)

Retinol (vitamin A) absorbed by cells is oxidized to retinal, which is further oxidized to RA in a reaction catalyzed mainly by ALDH1A1, ALDH1A2, and ALDH1A3 (24, 25). The metabolized RA product includes all-trans RA (ATRA), 9-cis RA, and 13-cis RA. Among these three forms, ALDH1 has higher affinity for ATRA and 9-cis RA, especially ALDH1A1 (19, 26). Canonically, RA suppresses tumor progression by binding to its nuclear receptors (RARα or RXR) and causes loss of stem cell markers, differentiation, cell cycle arrest, and morphology change (27). As a feedback loop, RA binds to RARα to inhibit ALDH1A1 through decreasing C/EBPβ (28, 29). On the other hand, RA has non-canonical pathways to promote tumor growth. RARs and RXRs form heterodimers with other receptors, such as estrogen receptor-α (ERα) and peroxisome proliferator-activated receptors (PPARβ/δ) (30, 31). The heterodimers of RXRs and ERα induce c-myc and cyclin D1, which promote tumor growth and inhibits apoptosis (22, 32). In addition, RXRs and PPARβ/δ form a dimer to upregulate pro-survival genes, including PDK1/Akt (19, 30, 33, 34). Thus, ALDH-mediated conversion of retinol to RA metabolites indirectly promotes prostate tumorigenesis and disease progression by converging on survival and tumor-promoting proliferative signaling pathways, such as Myc and PDK1/Akt1.

### WNT/β-catenin

ALDH1 was reported to be regulated by Wnt/β-catenin signaling in breast cancer (35), ovarian cancer (36), and PCa (37, 38). Typically, β-catenin degradation, induced by tankyrase inhibitor XAV939 or its siRNAs, decreases ALDH1A1 expression. Further biochemical analyses reveal that β-catenin/TCF transcriptional complex binds to the ALDH1A1 promoter *via* two consensus β-catenin/TCF recognition sites (37, 38). Therefore, inhibition of WNT signaling diminishes ALDH^high^ population and induces radiosensitization in PCa cells (39).

### TGF-β/Smad4

TGF-β/Smad4 pathway suppresses early tumor growth and promotes chemoresistance and bone metastasis in PCa (40–42). Furthermore, silence of SMAD4 upregulates the expression of ALDH1A1. Mechanistically, Dr. Hoshino et al. reveal that TGF-β regulates ALDH1A1 mRNA transcription through binding of Smad4 to its regulatory sequence (43). Thus, TGF-β/Smad4 pathway inhibits CSCs by transcriptional suppression of ALDH1A1.

### MUC1

Mucin 1 (MUC1) is a transmembrane protein aberrantly overexpressed in PCa (44). An increase in MUC1 gene copy number was observed in 35% of CRPCs (45). Dr. Alam et al. indicate that MUC1 induces ERK activation and thereby phosphorylates C/EBPβ. Furthermore, MUC1 and C/EBPβ form a transcriptional complex on the *ALDH1A1* gene promoter and activate *ALDH1A1* gene transcription (46). Collectively, MUC1 activates ERK and C/EBPβ pathways to increase ALDH^high^ CSCs.

### NOTCH

NOTCH controls cell differentiation in normal prostate gland and increased NOTCH signaling promotes PCa progression (47–50). It has been shown that NOTCH signaling regulates ALDH1A1 acetylation, which is negatively associated with ALDH activities. Dr. Zhao et al. indicate that p300/CBP-associated factor and deacetylase sirtuin 2 (SIRT2) are the enzymes responsible for the acetylation and deacetylation of ALDH1A1 K353. NOTCH activation results in SIRT2 induction, which deacetylates and activates ALDH1A1 (51). Therefore, NOTCH induces ALDH1A1 deacetylation to promote CSCs.

### Androgen receptor (AR)

AR signaling is a cornerstone of PCa progression and AR-targeted therapy is one of the most successful targeted therapies in cancer treatment (52, 53). In PCa cell line LNCaP, androgen dihydrotestosterone (DHT) induces ALDH1A3, but not ALDH1A1 and ALDH1A2. Mechanistically, the regulation of DHT on ALDH1A3 is likely due to the typical AR nuclear-translocation cascade (54). ALDH1A3 correlates with AR signaling pathway and localizes at luminal layers (55). Knockout of ALDH1A3 gradually acquired resistance to androgen deprivation therapy (56), although whether and how PCSCs are involved in this process remains unknown.

## Crucial regulators that maintain ALDH^high^ prostate CSCs

ALDH is a marker of cancer stem-like cells in different cancers, including PCa. Cancer stem-like cells are considered as a reservoir of cancer cells that can initiate tumor growth, survive after therapies, reestablish heterogeneous tumors, and thus cause drug resistance and reoccurrence (57). In addition to the signaling pathways that directly modulate ALDHs, accumulating efforts have been made to disclose prominent regulators in ALDH^high^ PCSCs. Here, we will endeavor to review recent findings regarding the pathways that regulate the maintenance of ALDH^high^ CSCs in PCa. Furthermore, applying the ALDH inhibitors for PCa therapies is potentially limited by the toxicity of pan-ALDH inhibitors and the redundancy of ALDH isoforms. Understanding crucial pathways that control the stemness of ALDH^high^ PCa cells would provide a valuable choice for developing novel therapeutic strategies for targeting PCSCs. We therefore summarize recent findings below and itemize key regulators in **Table 1**.

**Table 1:** Crucial signaling pathways that regulate ALDH+ cells in prostate cancer.

Involved signaling pathways	ALDH+ cells	Key genes	Gene functions in PCSC stemness	Findings in PCa research	References
WNT/β-Catenin	PC-3; DU 145	PER3	Suppress	Expression decreased in PCa	(20)
WNT/β-Catenin	C4-2B; DU 145	GSK3β	Suppress	Suppress PCa growth in soft tissue and bone	(58)
WNT/β-Catenin	PC-3	β-Catenin	Enhance	Suppress apoptosis in ALDH+ cells	(59)
EZH2/BRCA1	DU 145-RR; LNCaP-RR	BRCA1; EZH2	Suppress	Genetic silencing of EZH2 and BRCA1 enhances ALDH1A1 and ALDH1A3 activity	(60)
Glutamine/α-KG-	DU 145-RR; LNCaP-RR	Glutaminase	Enhance	Maintenance of the redox state, and enhanced radioresistance	(61)
SLUG/ TWIST1/SOX2	PC-3	TMPRSS4	Enhance	Promote cancer stem– like properties	(16)
PI3K/AKT	DU 145; LNCaP	HOXB9	Enhance	Alter the expression of a panel of CSC growth- and invasion/bone metastasis-related genes *via* TGF-β signaling	(62)
α_v_ and α6-integrins	PC-3M-Pro4Luc2; C4-2B	miR-25	Suppress	Expression in PCSCs is low/absent	(63)
Steroids/MMP9	PC-3M	cyclin A1/Aromatase	Enhance	Provide a suitable micro environment for PCSCs to establish bone metastatic growth	(64)
IL-6/STAT3	DU 145; LNCaP	STAT3	Enhance	Maintain the proportion of PCSCs and the expression of ALDH1A1	(65)

### WNT/β-catenin

Activation of the WNT/β-catenin pathway enhances ALDH transcription, and disruption of WNT signaling diminishes ALDH^high^ PCSCs (39). Furthermore, Period circadian regulator 3(PER3), a circadian rhythm gene, suppresses the WNT/β-catenin pathway *via* decreasing BMAL1 expression. Therefore, PER3 overexpression in ALDH^high^ cells significantly suppresses sphere formation and tumorigenicity (20). GSK3β phosphorylates β-catenin to reduce its nuclear localization. AR79, an inhibitor of GSK3β, increases the proportion of ALDH^high^ CD133+ cancer stem cell-like in PCa cell lines, promoting tumor growth and tumor-induced bone remodeling (58). Salinomycin, an antibiotic that selectively kills cancer stem-like cells, is also found to suppress WNT/β-catenin pathway and trigger more apoptosis in ALDH^high^ cells in PCa (59).

### BRCA1 and EZH2

BRCA1 negatively regulates PRC2-dependent H3K27 methylation, which involves multiple cellular processes, including DNA repair, transcriptional regulation, and DNA damage responses (66). EZH2, a core member of PRC2, promotes cancer progression by histone methylation-driven dedifferentiation (67). Silence of EZH2 and BRCA1 leads to a significant increase in the ALDH^high^ cell population, and knockdown of both genes has a cumulative effect on ALDH activity, suggesting that BRCA1 and EZH2 cooperate in the regulation of PCSCs phenotype (60).

### Glutaminase (GLS)

PCSCs are radioresistant and have a high glutamine demand. GLS-driven glutamine catabolism provides energy and maintains the redox state in cancer cells. Glutamine catabolism contributes to the maintenance of PCSCs by a α-KG dependent chromatin-modifying dioxygenase. Therefore, lack of glutamine decreases ALDH^high^ PCSCs and suppresses tumor formation in the xenograft mouse model (61).

### TMPRSS4

Transmembrane serine protease 4 (TMPRSS4) is a cell surface anchored serine protease that promotes resistance to anoikis, tumor sphere formation, and therapeutic resistance of PCa cells. TMPRSS4-induced invasive and metastatic phenotypes are accompanied by the upregulation of stemness factors, including SOX2, BMI1, and CD133. Importantly, TMPRSS4 increases ALDH^high^ PCSCs. Mechanistically, TMPRSS4 upregulates SLUG and TWIST1, which in turn increases the expression level of SOX2, a crucial stemness factor maintaining PCSCs (16).

### Human homeobox B9 (HOXB9)

HOXB9 is a transcription factor that is upregulated in PCa and found to be essential for PCa metastasis (68). HOXB9 alters cancer stem cell markers, such as CD44, and affects chemosensitivity and metastatic ability of ALDH^high^/CD44+/CXCR4+/CD24+ PCSCs (62). Mechanistically, silence of HOXB9 inhibits PCa cell proliferation and migration by suppressing PI3K/AKT pathways (68).

### miR-25

miR-25 expression is low or absent in PCSCs and increases during their differentiation into cells with a luminal epithelial phenotype. Furthermore, overexpression of miR-25 suppresses the migration and metastasis of ALDH^high^ cells by affecting the invasive cytoskeleton *via* directly targeting α_v_- and α_6_ integrins (63).

### Cyclin A1

Cyclin A1 is an important cell cycle regulator that is elevated in PCa (69). Interestingly, the expression of cyclin A1 is higher in metastatic lesions, such as lymph nodes, lung, and bone. Overexpression of cyclin A1 in ALDH^high^ PCa cells enhances bone metastatic growth and their self-renewal capability, suggesting that cyclin A1 is a key regulator of ALDH^high^ PCSCs. Furthermore, the expression of cyclin A1 is correlated with aromatase CYP19A1, which regulates androgen to estrogen metabolism. Estrogen and MMP9 facilitate the growth of ALDH^high^ PCSCs (64).

## Candidate ALDH targeted therapies in PCa

The crucial roles of ALDH validate that it is promising to target ALDH or ALDH^high^ cells to eradicate PCa. Recent years, multiple ALDH inhibitors have been developed for cancer therapy. Dr. Dinavahi et al. have made an elaborated review of ALDH inhibitors for cancer therapy (23). Herein, we focus on the ALDH inhibitors in PCa for their recent progress (**Supplementary Table 1**).

### 4-(Diethylamino)benzaldehyde (DEAB)

DEAB is a non-isoform-specific inhibitor commonly used for ALDH inhibitor in aldefluor assays (70). DEAB is a competitive inhibitor of ALDH1A1, 1A3, 1B1, and 5A1. Additionally, DEAB has been unraveled to irreversibly inactivates ALDH7A1 (71) and ALDH1A2 through forming a stable covalent adduct (72). In PCa, DEAB inhibits ALDH activity and ALDH^high^ population and decreases sphere-forming capability (73). However, the efficacy of DEAB was impeded by variations in ALDH expression. In addition, the lack of evidence in animal experiments also limits DEAB use for PCa treatment (74).

### DIMATE

DIMATE is an irreversible and competitive inhibitor of ALDH 1 and 3, suppressing ALDH activity and promoting apoptosis. In addition, the inhibition is reversibly for normal prostate cells but irreversible for PCa cells (75). Therefore, further animal and clinical trials are worth taking.

### Imidazo[1,2-a] pyridine derivatives

Imidazo[1,2-a] pyridine derivatives 3b and 6-(4-fluorophenyl)-2-phenylimidazo [1,2-a] pyridine inhibit ALDH1A1 and ALDH1A3 expression and suppress cell proliferation in different PCa cell lines. Derivative 3d more selectively inhibits colony formation of PC-3 PCa cells than PNT2-C2 or BPH-1 normal prostate epithelial cells (76, 77).

### Retinoic acid (RA)

A few evidence has proved RA as a potential therapy for PCa. Early in 2005, RA was found to reduce the activity of ALDH1A1 and ALDH3A1 and cause cytotoxicity (78). In addition, ALDH1A2, the enzyme responsible for RA synthesis, is reduced in PCa and associated with a shorter relapse-free survival (79). Furthermore, RA inhibits the proliferation and neuroendocrine phenotype of PCa cells and promote apoptosis in TRAMP model (80). However, a phase II trial of all trans retinoic acid (ATRA, a form of RA as we mentioned above) in hormone refractory PCa showed that patients did not respond to ATRA. This failure may be due to the low efficiency of drug delivery to tumor tissue or the rapid degradation of ATRA (81, 82). Accordingly, optimizing the delivery strategy of RA, such as the use of solid lipid nanoparticles, improves the efficiency of RA in tumor tissue (83). Moreover, RA affects multiple cellular pathways, which brings great challenges for RA use in clinical treatment. Currently, combined ATRA with 5-azacitidine (5-AZA) leads to an ongoing phase II clinical trial in PCa with PSA-only recurrence after local treatment (NCT03572387).

### Disulfiram (DSF)

DSF is well established as an alcohol abstinence drug by inhibiting acetaldehyde dehydrogenase (84). In addition, DSF also inhibits DNA topoisomerases (85) and DNA methylation (86), thus alleviating the proliferation of PCa cells. Strikingly, DSF selectively inhibits the growth of PCa cells at nanomolar concentrations compared to normal PrECs. However, DSF failed to completely block tumor growth of VCaP xenografts (86, 87) and change per-cell PSA level in a clinical trial (NCT01118741). Fortunately, the antiproliferation capability of DSF can be enhanced by copper or zinc in breast cancer and melanoma (88, 89). Accordingly, DSF/copper has been widely used to treat various cancers, including PCa. A phase Ib study with intravenous copper administration and oral DSF was performed in metastatic castration-resistant PCa. This clinical trial was terminated because oral administration of DSF showed poor stability and fast metabolism (NCT02963051) (90).

## Discussion

Accumulating studies have shown that ALDH is a promising stem cell marker for various CSCs, including PCa (91, 92). ALDH^high^ PCa cells have higher capabilities in self-renewal, clonogenicity and metastasis (12). ALDHs decrease oxidative stress for self-protection (93) and associate with less sensibility of tumor cells to chemotherapy and radiotherapy (94, 95). Especially, a high ALDH1A1 expression and ALDH3A1 is positively associated with a poor prognosis of PCa (21, 96). ALDH expression is regulated at the transcriptional level by RA signaling, WNT/β-catenin, and TGF-β. In addition, ALDH activity is modulated by NOTCH signaling *via* ALDH1A1 acetylation at K353 (**Figure 1**). Moreover, ALDHs are markers of cancer stem-like cells and regulate signaling transduction, such as canonical RA signaling, c-MYC, cyclinD1 and PDK1/AKT (**Figure 1**).

Compounds targeting ALDHs directly are still limited for PCa therapy (**Supplementary Table 1**). Collectively, potential reasons include: (1) toxicity of pan-ALDH inhibitors (23, 97, 98); (2) redundancy of ALDH isoforms (23, 99, 100); (3) Ineffective delivery method (81, 82, 101); (4) the complexity of RA signaling (102). As we summarized in **Figure 1**, RA has canonical and noncanonical pathways to suppress or promote tumor growth. Different concentrations of RA would have opposite functions in tumor progression (103). Therefore, targeting indispensable regulators in ALDH^high^ population instead would provide valuable options for eradicating PCSCs. We thus summarize crucial genes that control the bioactivities of ALDH^high^ PCa cells in **Table 1**. Some studies have tested some compounds focusing on WNT/β-catenin and STAT3 pathways, two prominent pathways that are essential for ALDH^high^ PCSCs (**Supplementary Table 1**). Galiellalactone (GL) inhibits the binding of activated STAT3 to DNA, reduces ALDH^high^ PCa population, downregulates ALDH1A1, and sensitizes chemotherapy (65, 104). Stattic binds to the SH2 domain of STAT3, inhibits its phosphorylation at Y705 (105), and reduces ALDH^high^ population in PC3M-1E8 and clinical PCa samples. Silibinin inhibits PCa cell proliferation and invasion by targeting STAT3 and WNT signaling (106). A clinical trial of Silibinin was performed, although no formal PSA response was detected and little silybin was found in PCa tissues (101). XAV-939, an effective tankyrase inhibitor antagonizing Wnt/β-catenin signaling (107), significantly reduces ALDH^high^ population, suppresses colonization and migration capacity of PCa cells, and sensitize PCa cells to radiotherapy (95).

Given CRPC is the lethal stage in PCa development, we ask if targeting ALDH could be effective for CRPC. Strikingly, STAT3 inhibitor Galiellalactone selectively promotes apoptosis in androgen-insensitive DU145 and PC-3 cells, but not in androgen-sensitive LNCaP cells, providing a potential approach for CRPC treatment (108). Therefore, targeting crucial regulators to disrupt the activity of ALDH^high^ population would provide promising strategy for CRPC treatment.

## Author contributions

MM summarized the regulators of ALDH+ prostate cancer cells; WH recapitulated the application and limitations for ALDH targeted therapies. KZ listed the signaling pathways regulated by ALDH. LX collected the expression levels of ALDHs in PCa. BZ and SX designed the overall frame, provided guidance, and revised and finalized the manuscript. All authors contributed to the article and approved the submitted version.

## Funding

This work was supported by grants 2021A1515110051 and 2021A1515110144 from GuangDong Basic and Applied Basic Research Foundation.

## Conflict of interest

The authors declare that the research was conducted in the absence of any commercial or financial relationships that could be construed as a potential conflict of interest.

## Publisher’s note

All claims expressed in this article are solely those of the authors and do not necessarily represent those of their affiliated organizations, or those of the publisher, the editors and the reviewers. Any product that may be evaluated in this article, or claim that may be made by its manufacturer, is not guaranteed or endorsed by the publisher.

## References

[1] Siegel RL Miller KD Fuchs HE Jemal A. Cancer statistic. CA Cancer J Clin (2022) 72:7–33. doi: 10.3322/caac.21708 35020204

[2] Loblaw DA Virgo KS Nam R Somerfield MR Ben-Josef E Mendelson DS. Initial hormonal management of androgen-sensitive metastatic, recurrent, or progressive prostate cancer: 2006 update of an American society of clinical oncology practice guideline. J Clin Oncol (2007) 25:1596–605. doi: 10.1200/JCO.2006.10.1949 17404365

[3] Dehm SM Schmidt LJ Heemers HV Vessella RL Tindall DJ. Splicing of a novel androgen receptor exon generates a constitutively active androgen receptor that mediates prostate cancer therapy resistance. Cancer Res (2008) 68:5469–77. doi: 10.1158/0008-5472.CAN-08-0594 PMC2663383 18593950

[4] Ramalingam S Ramamurthy VP Njar VCO. Dissecting major signaling pathways in prostate cancer development and progression: Mechanisms and novel therapeutic targets. J Steroid Biochem Mol Biol (2017) 166:16–27. doi: 10.1016/j.jsbmb.2016.07.006 27481707 PMC7371258

[5] Studer UE Hauri D Hanselmann S Chollet D Leisinger HJ Gasser T. Immediate versus deferred hormonal treatment for patients with prostate cancer who are not suitable for curative local treatment: results of the randomized trial SAKK 08/88. J Clin Oncol (2004) 22:4109–18. doi: 10.1200/JCO.2004.11.514 15483020

[6] Chandrasekar T Yang JC Gao AC Evans CP. Mechanisms of resistance in castration-resistant prostate cancer (CRPC). Transl Androl Urol (2015) 4:365–80. doi: 10.3978j.issn.2223-4683.2015.05.02 10.3978/j.issn.2223-4683.2015.05.02 PMC4708226 26814148

[7] Mansinho A Macedo D Fernandes I Costa L. Castration-resistant prostate cancer: Mechanisms, targets and treatment. Adv Exp Med Biol (2018) 1096:117–33. doi: 10.1007/978-3-319-99286-0_7 30324351

[8] Scher HI Fizazi K Saad F Taplin ME Sternberg CN Miller K. Increased survival with enzalutamide in prostate cancer after chemotherapy. N Engl J Med (2012) 367:1187–97. doi: 10.1056/NEJMoa1207506 22894553

[9] Dong L Zieren RC Xue W De Reijke TM Pienta KJ. Metastatic prostate cancer remains incurable, why? Asian J Urol (2019) 6:26–41. doi: 10.1016/j.ajur.2018.11.005 30775246 PMC6363601

[10] Chu P Clanton DJ Snipas TS Lee J Mitchell E Nguyen ML. Characterization of a subpopulation of colon cancer cells with stem cell-like properties. Int J Cancer (2009) 124:1312–21. doi: 10.1002/ijc.24061 19072981

[11] Patrawala L Calhoun T Schneider-Broussard R Li H Bhatia B Tang S. Highly purified CD44+ prostate cancer cells from xenograft human tumors are enriched in tumorigenic and metastatic progenitor cells. Oncogene (2006) 25:1696–708. doi: 10.1038/sj.onc.1209327 16449977

[12] Van Den Hoogen C van der Horst G Cheung H Buijs JT Lippitt JM Guzman-Ramirez N. High aldehyde dehydrogenase activity identifies tumor-initiating and metastasis-initiating cells in human prostate cancer. Cancer Res (2010) 70:5163–73. doi: 10.1158/0008-5472.CAN-09-3806 20516116

[13] Liu C Kelnar K Liu B Chen X Calhoun-Davis T Li H. The microRNA miR-34a inhibits prostate cancer stem cells and metastasis by directly repressing CD44. Nat Med (2011) 17:211–5. doi: 10.1038/nm.2284 PMC3076220 21240262

[14] Jeter CR Liu B Lu Y Chao HP Zhang D Liu X. NANOG reprograms prostate cancer cells to castration resistance *via* dynamically repressing and engaging the AR/FOXA1 signaling axis. Cell Discovery (2016) 2:16041. doi: 10.1038/celldisc.2016.41 27867534 PMC5109294

[15] Prager BC Xie Q Bao S Rich JN. Cancer stem cells: The architects of the tumor ecosystem. Cell Stem Cell (2019) 24:41–53. doi: 10.1016/j.stem.2018.12.009 30609398 PMC6350931

[16] Lee Y Yoon J Ko D Yu M Lee S Kim S. TMPRSS4 promotes cancer stem-like properties in prostate cancer cells through upregulation of SOX2 by SLUG and TWIST1. J Exp Clin Cancer Res (2021) 40:372. doi: 10.1186/s13046-021-02147-7 34809669 PMC8607621

[17] Tsao T Beretov J Ni J Bai X Bucci J Graham P. Cancer stem cells in prostate cancer radioresistance. Cancer Lett (2019) 465:94–104. doi: 10.1016/j.canlet.2019.08.020 31493443

[18] Püschel J Dubrovska A Gorodetska I. The multifaceted role of aldehyde dehydrogenases in prostate cancer stem cells. Cancers (Basel) (2021) 13:4703. doi: 10.3390/cancers13184703 34572930 PMC8472046

[19] Xu X Chai S Wang P Zhang C Yang Y Yang Y. Aldehyde dehydrogenases and cancer stem cells. Cancer Lett (2015) 369:50–7. doi: 10.1016/j.canlet.2015.08.018 26319899

[20] Li Q Xia D Wang Z Liu B Zhang J Peng P. Circadian rhythm gene PER3 negatively regulates stemness of prostate cancer stem cells *via* WNT/β-catenin signaling in tumor microenvironment. Front Cell Dev Biol (2021) 9:656981. doi: 10.3389/fcell.2021.656981 33816508 PMC8012816

[21] Yan J De Melo J Cutz JC Aziz T Tang D. Aldehyde dehydrogenase 3A1 associates with prostate tumorigenesis. Br J Cancer (2014) 110:2593–603. doi: 10.1038/bjc.2014.201 PMC4021532 24762960

[22] Tomita H Tanaka K Tanaka T Hara A. Aldehyde dehydrogenase 1A1 in stem cells and cancer. Oncotarget (2016) 7:11018–32. doi: 10.18632/oncotarget.6920 PMC4905455 26783961

[23] Dinavahi SS Bazewicz CG Gowda R Robertson GP. Aldehyde dehydrogenase inhibitors for cancer therapeutics. Trends Pharmacol Sci (2019) 40:774–89. doi: 10.1016/j.tips.2019.08.002 31515079

[24] Penzes P Wang X Napoli JL. Enzymatic characteristics of retinal dehydrogenase type I expressed in escherichia coli. Biochim Biophys Acta (1997) 1342:175–81. doi: 10.1016/S0167-4838(97)00102-7 9392526

[25] Black W Vasiliou V. The aldehyde dehydrogenase gene superfamily resource center. Hum Genomics (2009) 4:136–42. doi: 10.1186/1479-7364-4-2-136 PMC3525204 20038501

[26] Yoshida A Hsu LC Dave V. Retinal oxidation activity and biological role of human cytosolic aldehyde dehydrogenase. Enzyme (1992) 46:239–44. doi: 10.1159/000468794 1292933

[27] Ying M Wang S Sang Y Sun P Lal B Goodwin CR. Regulation of glioblastoma stem cells by retinoic acid: role for notch pathway inhibition. Oncogene (2011) 30:3454–67. doi: 10.1038/onc.2011.58 PMC3955956 21383690

[28] Yanagawa Y Chen JC Hsu LC Yoshida A. The transcriptional regulation of human aldehyde dehydrogenase I gene. the structural and functional analysis of the promoter. J Biol Chem (1995) 270:17521–7. doi: 10.1074/jbc.270.29.17521 7615557

[29] Elizondo G Corchero J Sterneck E Gonzalez FJ. Feedback inhibition of the retinaldehyde dehydrogenase gene ALDH1 by retinoic acid through retinoic acid receptor alpha and CCAAT/enhancer-binding protein beta. J Biol Chem (2000) 275:39747–53. doi: 10.1074/jbc.M004987200 10995752

[30] Schug TT Berry DC Toshkov IA Cheng L Nikitin AY Noy N. Overcoming retinoic acid-resistance of mammary carcinomas by diverting retinoic acid from PPARbeta/delta to RAR. Proc Natl Acad Sci U.S.A. (2008) 105:7546–51. doi: 10.1073/pnas.0709981105 PMC2396692 18495924

[31] Ross-Innes CS Stark R Holmes KA Schmidt D Spyrou C Russell R. Cooperative interaction between retinoic acid receptor-alpha and estrogen receptor in breast cancer. Genes Dev (2010) 24:171–82. doi: 10.1101/gad.552910 PMC2807352 20080953

[32] Chang Q Chen Z You J Mcnutt MA Zhang T Han Z. All-trans-retinoic acid induces cell growth arrest in a human medulloblastoma cell line. J Neurooncol (2007) 84:263–7. doi: 10.1007/s11060-007-9380-9 17453147

[33] Di-Poi N Tan NS Michalik L Wahli W Desvergne B. Antiapoptotic role of PPARbeta in keratinocytes *via* transcriptional control of the Akt1 signaling pathway. Mol Cell (2002) 10:721–33. doi: 10.1016/S1097-2765(02)00646-9 12419217

[34] Schug TT Berry DC Shaw NS Travis SN Noy N. Opposing effects of retinoic acid on cell growth result from alternate activation of two different nuclear receptors. Cell (2007) 129:723–33. doi: 10.1016/j.cell.2007.02.050 PMC1948722 17512406

[35] Jang GB Hong IS Kim RJ Lee SY Park SJ Lee ES. Wnt/β-catenin small-molecule inhibitor CWP232228 preferentially inhibits the growth of breast cancer stem-like cells. Cancer Res (2015) 75:1691–702. doi: 10.1158/0008-5472.CAN-14-2041 25660951

[36] Condello S Morgan CA Nagdas S Cao L Turek J Hurley TD. β-catenin-regulated ALDH1A1 is a target in ovarian cancer spheroids. Oncogene (2015) 34:2297–308. doi: 10.1038/onc.2014.178 PMC4275429 24954508

[37] Takahashi-Yanaga F Kahn M. Targeting wnt signaling: can we safely eradicate cancer stem cells? Clin Cancer Res (2010) 16:3153–62. doi: 10.1158/1078-0432.CCR-09-2943 20530697

[38] King TD Suto MJ Li Y. The wnt/β-catenin signaling pathway: a potential therapeutic target in the treatment of triple negative breast cancer. J Cell Biochem (2012) 113:13–8. doi: 10.1002/jcb.23350 PMC10924801 21898546

[39] Cojoc M Peitzsch C Kurth I Trautmann F Kunz-Schughart LA Telegeev GD. Aldehyde dehydrogenase is regulated by β-Catenin/TCF and promotes radioresistance in prostate cancer progenitor cells. Cancer Res (2015) 75:1482–94. doi: 10.1158/0008-5472.CAN-14-1924 25670168

[40] Ding Z Wu CJ Chu GC Xiao Y Ho D Zhang J. SMAD4-dependent barrier constrains prostate cancer growth and metastatic progression. Nature (2011) 470:269–73. doi: 10.1038/nature09677 PMC3753179 21289624

[41] Li Y Zhang B Xiang L Xia S Kucuk O Deng X. TGF-beta causes docetaxel resistance in prostate cancer *via* the induction of bcl-2 by acetylated KLF5 and protein stabilization. Theranostics (2020) 10:7656–70. doi: 10.7150/thno.44567 PMC7359077 32685011

[42] Zhang B Li Y Wu Q Xie L Barwick B Fu C. Acetylation of KLF5 maintains EMT and tumorigenicity to cause chemoresistant bone metastasis in prostate cancer. Nat Commun (2021) 12:1714. doi: 10.1038/s41467-021-21976-w 33731701 PMC7969754

[43] Hoshino Y Nishida J Katsuno Y Koinuma D Aoki T Kokudo N. Smad4 decreases the population of pancreatic cancer-initiating cells through transcriptional repression of ALDH1A1. Am J Pathol (2015) 185:1457–70. doi: 10.1016/j.ajpath.2015.01.011 25769430

[44] Kapoor A Gu Y Lin X Peng J Major P Tang D. MUCIN 1 in prostate cancer. In: Bott SRJ Ng KL, editors. Prostate cancer. (Brisbane AU: Exon Publications, Brisbane, Australia) (2021). 34181380

[45] Wong N Major P Kapoor A Wei F Yan J Aziz T. Amplification of MUC1 in prostate cancer metastasis and CRPC development. Oncotarget (2016) 7:83115–33. doi: 10.18632/oncotarget.13073 PMC5347757 27825118

[46] Alam M Ahmad R Rajabi H Kharbanda A Kufe D. MUC1-c oncoprotein activates ERK→C/EBPβ signaling and induction of aldehyde dehydrogenase 1A1 in breast cancer cells. J Biol Chem (2013) 288:30892–903. doi: 10.1074/jbc.M113.477158 PMC3829404 24043631

[47] Valdez JM Zhang L Su Q Dakhova O Zhang Y Shahi P. Notch and TGFbeta form a reciprocal positive regulatory loop that suppresses murine prostate basal stem/progenitor cell activity. Cell Stem Cell (2012) 11:676–88. doi: 10.1016/j.stem.2012.07.003 PMC3490134 23122291

[48] Kwon OJ Valdez JM Zhang L Zhang B Wei X Su Q. Increased notch signalling inhibits anoikis and stimulates proliferation of prostate luminal epithelial cells. Nat Commun (2014) 5:4416. doi: 10.1038/ncomms5416 25048699 PMC4167399

[49] Zhang B Ci X Tao R Ni JJ Xuan X King JL. Klf5 acetylation regulates luminal differentiation of basal progenitors in prostate development and regeneration. Nat Commun (2020) 11:997.68. doi: 10.1038/s41467-020-14737-8 32081850 PMC7035357

[50] Zhang B Xia S Liu M Li X Shuai S Tao W. Interruption of Klf5 acetylation in basal progenitor cells promotes luminal commitment by activating notch signaling. J Genet Genomics (2021). 49(6):579–82. doi: 10.1016/j.jgg.2021.11.013 34952235

[51] Zhao D Mo Y Li MT Zou SW Cheng ZL Sun YP. NOTCH-induced aldehyde dehydrogenase 1A1 deacetylation promotes breast cancer stem cells. J Clin Invest (2014) 124:5453–65. doi: 10.1172/JCI76611 PMC4348941 25384215

[52] Lokeshwar SD Klaassen Z Saad F. Treatment and trials in non-metastatic castration-resistant prostate cancer. Nat Rev Urol (2021) 18:433–42. doi: 10.1038/s41585-021-00470-4 34002069

[53] Wang Y Wang Y Ci X Choi SYC Crea F Lin D. Molecular events in neuroendocrine prostate cancer development. Nat Rev Urol (2021) 18:581–96. doi: 10.1038/s41585-021-00490-0 PMC10802813 34290447

[54] Trasino SE Harrison EH Wang TT. Androgen regulation of aldehyde dehydrogenase 1A3 (ALDH1A3) in the androgen-responsive human prostate cancer cell line LNCaP. Exp Biol Med (Maywood) (2007) 232:762–71. doi: 10.3181/00379727-232-232076 17526768

[55] Wang S Liang C Bao M Li X Zhang L Li S. ALDH1A3 correlates with luminal phenotype in prostate cancer. Tumour Biol (2017) 39:1010428317703652. doi: 10.1177/1010428317703652 28443495

[56] Wang S Zhou X Liang C Bao M Tian Y Zhu J. ALDH1A3 serves as a predictor for castration resistance in prostate cancer patients. BMC Cancer (2020) 20:387. doi: 10.1186/s12885-020-06899-x 32375698 PMC7201787

[57] Kushwaha PP Verma S Kumar S Gupta S. Role of prostate cancer stem-like cells in the development of antiandrogen resistance. Cancer Drug Resist (2022) 5:459–71. doi: 10.20517/cdr.2022.07 PMC9255247 35800367

[58] Jiang Y Dai J Zhang H Sottnik JL Keller JM Escott KJ. Activation of the wnt pathway through AR79, a GSK3β inhibitor, promotes prostate cancer growth in soft tissue and bone. Mol Cancer Res (2013) 11:1597–610. doi: 10.1158/1541-7786.MCR-13-0332-T PMC3869871 24088787

[59] Zhang Y Liu L Li F Wu T Jiang H Jiang X. Salinomycin exerts anticancer effects on PC-3 cells and PC-3-Derived cancer stem cells *In vitro* and *In vivo* . BioMed Res Int (2017) 2017:4101653. doi: 10.1155/2017/4101653 28676857 PMC5476894

[60] Gorodetska I Lukiyanchuk V Peitzsch C Kozeretska I Dubrovska A. BRCA1 and EZH2 cooperate in regulation of prostate cancer stem cell phenotype. Int J Cancer (2019) 145:2974–85. doi: 10.1002/ijc.32323 30968962

[61] Mukha A Kahya U Linge A Chen O Löck S Lukiyanchuk V. GLS-driven glutamine catabolism contributes to prostate cancer radiosensitivity by regulating the redox state, stemness and ATG5-mediated autophagy. Theranostics (2021) 11:7844–68. doi: 10.7150/thno.58655 PMC8315064 34335968

[62] Sui Y Hu W Zhang W Li D Zhu H You Q. Insights into homeobox B9: a propeller for metastasis in dormant prostate cancer progenitor cells. Br J Cancer (2021) 125:1003–15. doi: 10.1038/s41416-021-01482-y PMC8476533 34247196

[63] Zoni E van der Horst G Van De Merbel AF Chen L Rane JK Pelger RC. miR-25 modulates invasiveness and dissemination of human prostate cancer cells *via* regulation of αv- and α6-integrin expression. Cancer Res (2015) 75:2326–36. doi: 10.1158/0008-5472.CAN-14-2155 25858144

[64] Miftakhova R Hedblom A Semenas J Robinson B Simoulis A Malm J. Cyclin A1 and P450 aromatase promote metastatic homing and growth of stem-like prostate cancer cells in the bone marrow. Cancer Res (2016) 76:2453–64. doi: 10.1158/0008-5472.CAN-15-2340 26921336

[65] Hellsten R Johansson M Dahlman A Sterner O Bjartell A. Galiellalactone inhibits stem cell-like ALDH-positive prostate cancer cells. PloS One (2011) 6:e22118. doi: 10.1371/journal.pone.0022118 21779382 PMC3133629

[66] Gorodetska I Kozeretska I Dubrovska A. BRCA genes: The role in genome stability, cancer stemness and therapy resistance. J Cancer (2019) 10:2109–27. doi: 10.7150/jca.30410 PMC6548160 31205572

[67] Ruggero K Farran-Matas S Martinez-Tebar A Aytes A. Epigenetic regulation in prostate cancer progression. Curr Mol Biol Rep (2018) 4:101–15. doi: 10.1007/s40610-018-0095-9 PMC5976687 29888169

[68] Xu H Wu S Shen X Wu D Qin Z Wang H. Silencing of HOXB9 suppresses cellular proliferation, angiogenesis, migration and invasion of prostate cancer cells. J Biosci (2020) 45. doi: 10.1007/s12038-020-0013-1 32098919

[69] Wegiel B Bjartell A Tuomela J Dizeyi N Tinzl M Helczynski L. Multiple cellular mechanisms related to cyclin A1 in prostate cancer invasion and metastasis. J Natl Cancer Inst (2008) 100:1022–36. doi: 10.1093/jnci/djn214 PMC2467435 18612129

[70] Russo J Chung S Contreras K Lian B Lorenz J Stevens D. Identification of 4-(N,N-Dipropylamino)Benzaldehyde as a potent, reversible inhibitor of mouse and human class-I aldehyde dehydrogenase. Biochem Pharmacol (1995) 50:399–406. doi: 10.1016/0006-2952(95)00138-P 7646541

[71] Luo M Gates KS Henzl MT Tanner JJ. Diethylaminobenzaldehyde is a covalent, irreversible inactivator of ALDH7A1. ACS Chem Biol (2015) 10:693–7. doi: 10.1021/cb500977q 25554827

[72] Morgan CA Parajuli B Buchman CD Dria K Hurley TD. N,N-diethylaminobenzaldehyde (DEAB) as a substrate and mechanism-based inhibitor for human ALDH isoenzymes. Chemico-Biological Interact (2015) 234:18–28. doi: 10.1016/j.cbi.2014.12.008 PMC4414715 25512087

[73] Gangavarapu KJ Azabdaftari G Morrison CD Miller A Foster BA Huss WJ. Aldehyde dehydrogenase and ATP binding cassette transporter G2 (ABCG2) functional assays isolate different populations of prostate stem cells where ABCG2 function selects for cells with increased stem cell activity. Stem Cell Res Ther (2013) 4:132. doi: 10.1186/scrt343 24405526 PMC3854760

[74] Matsunaga N Ogino T Hara Y Tanaka T Koyanagi S Ohdo S. Optimized dosing schedule based on circadian dynamics of mouse breast cancer stem cells improves the antitumor effects of aldehyde dehydrogenase inhibitor. Cancer Res (2018) 78:3698–708. doi: 10.1158/0008-5472.CAN-17-4034 29735553

[75] Quash G Fournet G Courvoisier C Martinez RM Chantepie J Paret MJ. Aldehyde dehydrogenase inhibitors: alpha,beta-acetylenic n-substituted aminothiolesters are reversible growth inhibitors of normal epithelial but irreversible apoptogens for cancer epithelial cells from human prostate in culture. Eur J Medicinal Chem (2008) 43:906–16. doi: 10.1016/j.ejmech.2007.06.004 17692435

[76] Quattrini L Gelardi ELM Coviello V Sartini S Ferraris DM Mori M. Imidazo[1,2-a]pyridine derivatives as aldehyde dehydrogenase inhibitors: Novel chemotypes to target glioblastoma stem cells. J Medicinal Chem (2020) 63:4603–16. doi: 10.1021/acs.jmedchem.9b01910 32223240

[77] Quattrini L Sadiq M Petrarolo G Maitland NJ Frame FM Pors K. Aldehyde dehydrogenases and prostate cancer: Shedding light on isoform distribution to reveal druggable target. Biomedicines (2020) 8:569. doi: 10.3390/biomedicines8120569 PMC7761903 33291762

[78] Moreb JS Gabr A Vartikar GR Gowda S Zucali JR Mohuczy D. Retinoic acid down-regulates aldehyde dehydrogenase and increases cytotoxicity of 4-hydroperoxycyclophosphamide and acetaldehyde. J Pharmacol Exp Ther (2005) 312:339–45. doi: 10.1124/jpet.104.072496 15470086

[79] Kim H Lapointe J Kaygusuz G Ong DE Li C Van De Rijn M. The retinoic acid synthesis gene ALDH1a2 is a candidate tumor suppressor in prostate cancer. Cancer Res (2005) 65:8118–24. doi: 10.1158/0008-5472.CAN-04-4562 16166285

[80] Huss WJ Lai LH Barrios RJ Hirschi KK Greenberg NM. Retinoic acid slows progression and promotes apoptosis of spontaneous prostate cancer. Prostate (2004) 61:142–52. doi: 10.1002/pros.20097 15305337

[81] Trump DL Smith DC Stiff D Adedoyin A Day R Bahnson RR. A phase II trial of all-trans-retinoic acid in hormone-refractory prostate cancer: a clinical trial with detailed pharmacokinetic analysis. Cancer Chemother Pharmacol (1997) 39:349–56. doi: 10.1007/s002800050582 9025776

[82] White JA Beckett-Jones B Guo YD Dilworth FJ Bonasoro J Jones G. cDNA cloning of human retinoic acid-metabolizing enzyme (hP450RAI) identifies a novel family of cytochromes P450. J Biol Chem (1997) 272:18538–41. doi: 10.1074/jbc.272.30.18538 9228017

[83] Akanda MH Rai R Slipper IJ Chowdhry BZ Lamprou D Getti G. Delivery of retinoic acid to LNCap human prostate cancer cells using solid lipid nanoparticles. Int J Pharmaceutics (2015) 493:161–71. doi: 10.1016/j.ijpharm.2015.07.042 26200751

[84] Veverka KA Johnson KL Mays DC Lipsky JJ Naylor S. Inhibition of aldehyde dehydrogenase by disulfiram and its metabolite methyl diethylthiocarbamoyl-sulfoxide. Biochem Pharmacol (1997) 53:511–8. doi: 10.1016/S0006-2952(96)00767-8 9105402

[85] Yakisich JS Siden A Eneroth P Cruz M. Disulfiram is a potent *in vitro* inhibitor of DNA topoisomerases. Biochem Biophys Res Commun (2001) 289:586–90. doi: 10.1006/bbrc.2001.6027 11716515

[86] Lin J Haffner MC Zhang Y Lee BH Brennen WN Britton J. Disulfiram is a DNA demethylating agent and inhibits prostate cancer cell growth. Prostate (2011) 71:333–43. doi: 10.1002/pros.21247 PMC3043358 20809552

[87] Iljin K Ketola K Vainio P Halonen P Kohonen P Fey V. High-throughput cell-based screening of 4910 known drugs and drug-like small molecules identifies disulfiram as an inhibitor of prostate cancer cell growth. Clin Cancer Res (2009) 15:6070–8. doi: 10.1158/1078-0432.CCR-09-1035 19789329

[88] Brar SS Grigg C Wilson KS Holder WD Dreau D Austin C. Disulfiram inhibits activating transcription factor/cyclic AMP-responsive element binding protein and human melanoma growth in a metal-dependent manner *in vitro*, in mice and in a patient with metastatic disease. Mol Cancer Ther (2004) 3:1049–60. doi: 10.1158/1535-7163.1049.3.9 15367699

[89] Chen D Cui QZC Yang HJ Dou QP. Disulfiram, a clinically used anti-alcoholism drug and copper-binding agent, induces apoptotic cell death in breast cancer cultures and xenografts *via* inhibition of the proteasome activity. Cancer Res (2006) 66:10425–33. doi: 10.1158/0008-5472.CAN-06-2126 17079463

[90] Zhang T Kephart J Bronson E Anand M Daly C Spasojevic I. Disulfiram (DSF) pharmacokinetics (PK) and copper PET imaging in a phase ib study of intravenous (IV) copper loading with oral DSF for patients with metastatic castration-resistant prostate cancer (mCRPC). J Clin Oncol (2020) 38:96. doi: 10.1200/JCO.2020.38.6_suppl.96

[91] Tang DG Patrawala L Calhoun T Bhatia B Choy G Schneider-Broussard R. Prostate cancer stem/progenitor cells: identification, characterization, and implications. Mol Carcinog (2007) 46:1–14. doi: 10.1002/mc.20255 16921491

[92] Clark DW Palle K. Aldehyde dehydrogenases in cancer stem cells: potential as therapeutic targets. Ann Transl Med (2016) 4:518. doi: 10.21037/atm.2016.11.82 28149880 PMC5233526

[93] Singh S Brocker C Koppaka V Chen Y Jackson BC Matsumoto A. Aldehyde dehydrogenases in cellular responses to oxidative/electrophilic stress. Free Radic Biol Med (2013) 56:89–101. doi: 10.1016/j.freeradbiomed.2012.11.010 23195683 PMC3631350

[94] Sladek NE. Aldehyde dehydrogenase-mediated cellular relative insensitivity to the oxazaphosphorines. Curr Pharm Des (1999) 5:607–25. 10469894

[95] Cojoc M Peitzsch C Kurth I Trautmann F Kunz-Schughart LA Telegeev GD. Aldehyde dehydrogenase is regulated by beta-Catenin/TCF and promotes radioresistance in prostate cancer progenitor cells. Cancer Res (2015) 75:1482–94. doi: 10.1158/0008-5472.CAN-14-1924 25670168

[96] Li T Su Y Mei Y Leng Q Leng B Liu Z. ALDH1A1 is a marker for malignant prostate stem cells and predictor of prostate cancer patients' outcome. Lab Invest (2010) 90:234–44. doi: 10.1038/labinvest.2009.127 PMC3552330 20010854

[97] Wang MF Han CL Yin SJ. Substrate specificity of human and yeast aldehyde dehydrogenases. Chem Biol Interact (2009) 178:36–9. doi: 10.1016/j.cbi.2008.10.002 18983993

[98] Koppaka V Thompson DC Chen Y Ellermann M Nicolaou KC Juvonen RO. Aldehyde dehydrogenase inhibitors: a comprehensive review of the pharmacology, mechanism of action, substrate specificity, and clinical application. Pharmacol Rev (2012) 64:520–39. doi: 10.1124/pr.111.005538 PMC3400832 22544865

[99] Sladek NE. Human aldehyde dehydrogenases: potential pathological, pharmacological, and toxicological impact. J Biochem Mol Toxicol (2003) 17:7–23. doi: 10.1002/jbt.10057 12616643

[100] Morgan CA Hurley TD. Development of a high-throughput *in vitro* assay to identify selective inhibitors for human ALDH1A1. Chem Biol Interact (2015) 234:29–37. doi: 10.1016/j.cbi.2014.10.028 25450233 PMC4414680

[101] Flaig TW Gustafson DL Su LJ Zirrolli JA Crighton F Harrison GS. A phase I and pharmacokinetic study of silybin-phytosome in prostate cancer patients. Investigational New Drugs (2007) 25:139–46. doi: 10.1007/s10637-006-9019-2 17077998

[102] Di Masi A Leboffe L De Marinis E Pagano F Cicconi L Rochette-Egly C. Retinoic acid receptors: from molecular mechanisms to cancer therapy. Mol Aspects Med (2015) 41:1–115. doi: 10.1016/j.mam.2014.12.003 25543955

[103] Petrie K Urban-Wojciuk Z Sbirkov Y Graham A Hamann A Brown G. Retinoic acid receptor gamma is a therapeutically targetable driver of growth and survival in prostate cancer. Cancer Rep (Hoboken) (2020) 3:e1284. doi: 10.1002/cnr2.1284 32881426 PMC7941583

[104] Canesin G Maggio V Palominos M Stiehm A Contreras HR Castellon EA. STAT3 inhibition with galiellalactone effectively targets the prostate cancer stem-like cell population. Sci Rep (2020) 10:1395. doi: 10.1038/s41598-020-70948-5 32811873 PMC7434889

[105] Schust J Sperl B Hollis A Mayer TU Berg T. Stattic: A small-molecule inhibitor of STAT3 activation and dimerization. Chem Biol (2006) 13:1235–42. doi: 10.1016/j.chembiol.2006.09.018 17114005

[106] Jiang Y Song H Jiang L Qiao Y Yang D Wang D. Silybin prevents prostate cancer by inhibited the ALDH1A1 expression in the retinol metabolism pathway. Front Cell Dev Biol (2020) 8:574394. doi: 10.3389/fcell.2020.574394 32984354 PMC7487981

[107] Huang SMA Mishina YM Liu SM Cheung A Stegmeier F Michaud GA. Tankyrase inhibition stabilizes axin and antagonizes wnt signalling. Nature (2009) 461:614–20. doi: 10.1038/nature08356 19759537

[108] Hellsten R Johansson M Dahlman A Dizeyi N Sterner O Bjartell A. Galiellalactone is a novel therapeutic candidate against hormone-refractory prostate cancer expressing activated Stat3. Prostate (2008) 68:269–80. doi: 10.1002/pros.20699 18163422

