# Supplementary material for: Targeting aldehyde dehydrogenase for prostate cancer therapies
Source: Front Oncol. 2022 Oct 10;12:1006340. doi: 10.3389/fonc.2022.1006340 (PMC9589344; doi:10.3389/fonc.2022.1006340)
Supplement: Supplementary file 1 [file DataSheet_1.pdf]

Supplementary Table 1. Compounds targeting ALDH in prostate cancer

Agent	Targets	Cellular activity	PCa cell lines	Clinic trial in PCa	Reference
**Directly targeting ALDH in prostate cancer**					
N, N-diethylaminobenzaldehyde (DEAB)	Competitive inhibition of ALDH1A1, 1A3, 1B1, and 5A1, irreversibly inactivates ALDH7A1, ALDH1A2 and 2	Inhibit ALDH activity and ALDH^{high} population, decrease sphere-forming capability in CWR-R1 cell lines	RWPE-2, CWR-R1 and DU 145	Not available	(Gangavarapu et al., 2013)
4-Dimethylamino-4-methyl-pent-2-ynthioic acid-S-methylester (DIMATE)	Competitive and irreversible ALDH inhibition, targeting ALDH1 and ALDH3	Suppress ALDH activity, the inhibition of cell growth is reversible for normal epithelial cells but irreversible for prostate cancer epithelial cells	DU 145, MOP and ME， HPENC	Not available	(Quash et al., 2008)
Imidazo [1,2-a] pyridine derivatives	Primarily target ALDH1A1 and ALDH1A3	Inhibit ALDH1A1 and ALDH1A3 gene expression, and proliferation in normal prostate epithelial cell line PNT2-C2, benign prostatic hyperplasia cell line BPH1, and cancer cell line PC-3	PNT2-C2; BPH1; PC-3, LNCaP, P4E6; H796/19， H798/19	Not available	(Le Magnen et al., 2013;Quattrini et al., 2020a;Quattrini et al., 2020b)
Retinoic acid	ALDH1A1 and ALDH3A1	Decrease ALDH1A1 and ALDH3A1 activity and drive differentiation of CSCs	PNT1A, PC-3, LNCaP, P4E6, BPH1, Primary cultures derived from patient prostate tissues	NCT03572387 (Phase 2)	(Pollard et al., 1991;Huss et al., 2004;Moreb et al., 2005;Young et al., 2015;Seed et al., 2019)
Disulfiram (DSF/DS)	Irreversibly inhibit the ALDH (via covalent modification of the active site)	Selectively inhibit proliferation of prostate cancer cell compared to normal PrECs	VCaP, DUCaP, LNCaP, LNCaP C4-2, DU 145, PC-3, EP156T, RWPE-1 and PrEC cells	NCT01118741 (Not Applicable)<br><br>NCT02963051 (Phase 1)	(Veverka et al., 1997;Iljin et al., 2009;Lin et al., 2011)
**Targeting crucial regulators in ALDH^{high} population in prostate cancer**					
Galiellalactone	STAT3 pathway	Downregulate gene expression of ALDH1A1, reduce ALDH^{high} stem cell-like population, and induce its apoptosis	DU 145, PC-3, LNCaP	Not available	(Hellsten et al., 2011;Canesin et al., 2020)
Stattic		Reduce ALDH^{high} cell population (with high phosphorylated STAT3 levels)	PC-3M-1E8, clinic samples	Not available	(Schust et al., 2006;Han et al., 2014)
Silibinin	Constitutively inactivate STAT-3, Wnt/β-catenin pathway	Downregulate ALDH1A1 expression, inhibit cell migration, invasion, and growth of ALDH^{high} cells	DU 145, PC-3 cells	NCT00487721 (Phase 2)	(Singh et al., 2002;Agarwal et al., 2007;Lu et al., 2012;Jiang et al., 2020)
XAV939	WNT/β-catenin signaling pathway	Reduce the expression of ALDH1A1 (especially in PC-3) and ALDH^{high} population, making it sensitive to radiotherapy	DU 145, C4-2B, PC-3 and LNCaP	Not available	(Cojoc et al., 2015)

Reference

Agarwal, C., Tyagi, A., Kaur, M., and Agarwal, R. (2007). Silibinin inhibits constitutive activation of Stat3, and causes caspase activation and apoptotic death of human prostate carcinoma DU145 cells. *Carcinogenesis* 28**,** 1463-1470.

Canesin, G., Maggio, V., Palominos, M., Stiehm, A., Contreras, H.R., Castellon, E.A., Morote, J., Paciucci, R., Maitland, N.J., Bjartell, A., and Hellsten, R. (2020). STAT3 inhibition with galiellalactone effectively targets the prostate cancer stem-like cell population. *Scientific Reports* 10.

Cojoc, M., Peitzsch, C., Kurth, I., Trautmann, F., Kunz-Schughart, L.A., Telegeev, G.D., Stakhovsky, E.A., Walker, J.R., Simin, K., Lyle, S., Fuessel, S., Erdmann, K., Wirth, M.P., Krause, M., Baumann, M., and Dubrovska, A. (2015). Aldehyde Dehydrogenase Is Regulated by beta-Catenin/TCF and Promotes Radioresistance in Prostate Cancer Progenitor Cells. *Cancer Res* 75**,** 1482-1494.

Gangavarapu, K.J., Azabdaftari, G., Morrison, C.D., Miller, A., Foster, B.A., and Huss, W.J. (2013). Aldehyde dehydrogenase and ATP binding cassette transporter G2 (ABCG2) functional assays isolate different populations of prostate stem cells where ABCG2 function selects for cells with increased stem cell activity. *Stem Cell Research & Therapy* 4.

Han, Z.Q., Wang, X.L., Ma, L., Chen, L.J., Xiao, M., Huang, L., Cao, Y., Bai, J., Ma, D., Zhou, J.F., and Hong, Z.Y. (2014). Inhibition of STAT3 signaling targets both tumor-initiating and differentiated cell populations in prostate cancer. *Oncotarget* 5**,** 8416-8428.

Hellsten, R., Johansson, M., Dahlman, A., Sterner, O., and Bjartell, A. (2011). Galiellalactone Inhibits Stem Cell-Like ALDH-Positive Prostate Cancer Cells. *Plos One* 6.

Huss, W.J., Lai, L.H., Barrios, R.J., Hirschi, K.K., and Greenberg, N.M. (2004). Retinoic acid slows progression and promotes apoptosis of spontaneous prostate cancer. *Prostate* 61**,** 142-152.

Iljin, K., Ketola, K., Vainio, P., Halonen, P., Kohonen, P., Fey, V., Grafstrom, R.C., Perala, M., and Kallioniemi, O. (2009). High-Throughput Cell-Based Screening of 4910 Known Drugs and Drug-like Small Molecules Identifies Disulfiram as an Inhibitor of Prostate Cancer Cell Growth. *Clinical Cancer Research* 15**,** 6070-6078.

Jiang, Y., Song, H., Jiang, L., Qiao, Y., Yang, D., Wang, D., and Li, J. (2020). Silybin Prevents Prostate Cancer by Inhibited the ALDH1A1 Expression in the Retinol Metabolism Pathway. *Front Cell Dev Biol* 8**,** 574394.

Le Magnen, C., Bubendorf, L., Rentsch, C.A., Mengus, C., Gsponer, J., Zellweger, T., Rieken, M., Thalmann, G.N., Cecchini, M.G., Germann, M., Bachmann, A., Wyler, S., Heberer, M., and Spagnoli, G.C. (2013). Characterization and clinical relevance of ALDHbright populations in prostate cancer. *Clin Cancer Res* 19**,** 5361-5371.

Lin, J., Haffner, M.C., Zhang, Y., Lee, B.H., Brennen, W.N., Britton, J., Kachhap, S.K., Shim, J.S., Liu, J.O., Nelson, W.G., Yegnasubramanian, S., and Carducci, M.A. (2011). Disulfiram is a DNA demethylating agent and inhibits prostate cancer cell growth. *Prostate* 71**,** 333-343.

Lu, W.Y., Lin, C.H., King, T.D., Chen, H.H., Reynolds, R.C., and Li, Y.H. (2012). Silibinin inhibits Wnt/beta-catenin signaling by suppressing Wnt co-receptor LRP6 expression in human prostate and breast cancer cells. *Cellular Signalling* 24**,** 2291-2296.

Moreb, J.S., Gabr, A., Vartikar, G.R., Gowda, S., Zucali, J.R., and Mohuczy, D. (2005). Retinoic acid down-regulates aldehyde dehydrogenase and increases cytotoxicity of 4-hydroperoxycyclophosphamide and acetaldehyde. *Journal of Pharmacology and Experimental Therapeutics* 312**,** 339-345.

Pollard, M., Luckert, P.H., and Sporn, M.B. (1991). Prevention of Primary Prostate-Cancer in Lobund-Wistar Rats by N-(4-Hydroxyphenyl)Retinamide. *Cancer Research* 51**,** 3610-3611.

Quash, G., Fournet, G., Courvoisier, C., Martinez, R.M., Chantepie, J., Paret, M.J., Pharaboz, J., Joly-Pharaboz, M.O., Gore, J., Andre, J., and Reichert, U. (2008). Aldehyde dehydrogenase inhibitors:

alpha,beta-Acetylenic N-substituted aminothiolesters are reversible growth inhibitors of normal epithelial but irreversible apoptogens for cancer epithelial cells from human prostate in culture. *European Journal of Medicinal Chemistry* 43**,** 906-916.

Quattrini, L., Gelardi, E.L.M., Coviello, V., Sartini, S., Ferraris, D.M., Mori, M., Nakano, I., Garavaglia, S., and La Motta, C. (2020a). Imidazo[1,2-a]pyridine Derivatives as Aldehyde Dehydrogenase Inhibitors: Novel Chemotypes to Target Glioblastoma Stem Cells. *Journal of Medicinal Chemistry* 63**,** 4603-4616.

Quattrini, L., Sadiq, M., Petrarolo, G., Maitland, N.J., Frame, F.M., Pors, K., and La Motta, C. (2020b). Aldehyde Dehydrogenases and Prostate Cancer: Shedding Light on Isoform Distribution to Reveal Druggable Target. *Biomedicines* 8.

Schust, J., Sperl, B., Hollis, A., Mayer, T.U., and Berg, T. (2006). Stattic: A small-molecule inhibitor of STAT3 activation and dimerization. *Chemistry & Biology* 13**,** 1235-1242.

Seed, R.I., Taurozzi, A.J., Wilcock, D.J., Nappo, G., Erb, H.H.H., Read, M.L., Gurney, M., Archer, L.K., Ito, S., Rumsby, M.G., Petrie, J.L., Clayton, A., Maitland, N.J., and Collins, A.T. (2019). The putative tumour suppressor protein Latexin is secreted by prostate luminal cells and is downregulated in malignancy. *Scientific Reports* 9.

Singh, R.P., Dhanalakshmi, S., Tyagi, A.K., Chan, D.C.F., Agarwal, C., and Agarwal, R. (2002). Dietary feeding of silibinin inhibits advance human prostate carcinoma growth in athymic nude mice and increases plasma insulin-like growth factor-binding protein-3 levels. *Cancer Research* 62**,** 3063-3069.

Veverka, K.A., Johnson, K.L., Mays, D.C., Lipsky, J.J., and Naylor, S. (1997). Inhibition of aldehyde dehydrogenase by disulfiram and its metabolite methyl diethylthiocarbamoyl-sulfoxide. *Biochemical Pharmacology* 53**,** 511-518.

Young, M.J., Wu, Y.H., Chiu, W.T., Weng, T.Y., Huang, Y.F., and Chou, C.Y. (2015). All-trans retinoic acid downregulates ALDH1-mediated stemness and inhibits tumour formation in ovarian cancer cells. *Carcinogenesis* 36**,** 498-507.
